# The Role of Clopidogrel in 2020: A Reappraisal

**DOI:** 10.1155/2020/8703627

**Published:** 2020-03-16

**Authors:** Giuseppe Patti, Giuseppe Micieli, Claudio Cimminiello, Leonardo Bolognese

**Affiliations:** ^1^Dipartimento Universitario di Medicina Traslazionale, Università Piemonte Orientale, Azienda Ospedaliero-Universitaria Maggiore della Carità di Novara, Novara, Italy; ^2^Dipartimento di Neurologia d'Urgenza, IRCCS Fondazione Istituto Neurologico Nazionale C. Mondino, Pavia, Italy; ^3^Studies and Research Center of the Italian Society of Angiology and Vascular Pathology (Società Italiana di Angiologia e Patologia Vascolare, SIAPAV), Milan, Italy; ^4^Dipartimento Cardio Neuro Vascolare, Ospedale, San Donato, Arezzo, Italy

## Abstract

Antiplatelet therapy is the mainstay of treatment and secondary prevention of cardiovascular disease (CVD), including acute coronary syndrome (ACS), transient ischemic attack (TIA) or minor stroke, and peripheral artery disease (PAD). The P2Y_12_ inhibitors, of which clopidogrel was the first, play an integral role in antiplatelet therapy and therefore in the treatment and secondary prevention of CVD. This review discusses the available evidence concerning antiplatelet therapy in patients with CVD, with a focus on the role of clopidogrel. In combination with aspirin, clopidogrel is often used as part of dual antiplatelet therapy (DAPT) for the secondary prevention of ACS. Although newer, more potent P2Y_12_ inhibitors (prasugrel and ticagrelor) show a greater reduction in ischemic risk compared with clopidogrel in randomized trials of ACS patients, these newer P2Y_12_ inhibitors are often associated with an increased risk of bleeding. Deescalation of DAPT by switching from prasugrel or ticagrelor to clopidogrel may be required in some patients with ACS. Furthermore, real-world studies of ACS patients have not confirmed the benefits of the newer P2Y_12_ inhibitors over clopidogrel. In patients with very high-risk TIA or stroke, short-term DAPT with clopidogrel plus aspirin for 21–28 days, followed by clopidogrel monotherapy for up to 90 days, is recommended. Clopidogrel monotherapy may also be used in patients with symptomatic PAD. In conclusion, there is strong evidence supporting the use of clopidogrel antiplatelet therapy in several clinical settings, which emphasizes the importance of this medication in clinical practice.

## 1. Introduction

There has been an increase in the incidence and prevalence of cardiovascular disease (CVD) in the past few decades, including acute coronary syndrome (ACS), which has become a leading cause of mortality and morbidity worldwide [1–5]. The number of CVD-related deaths has increased by 12.5% during the past decade, accounting for approximately one-third of all deaths globally, mainly because of population growth and aging [3]. Patients with ACS have an increased risk of new ischemic events [6, 7], and ischemic heart disease and stroke are main contributors to global CVD burden [3].

In patients with CVD, platelet activation is triggered by an injured or dysfunctional vascular endothelium, which leads to platelet aggregation and subsequent pathologic thrombus formation and ischemic events [8]. Hence, antiplatelet therapy is the mainstay of the treatment and secondary prevention of CVD. The first medication used as an antiplatelet agent was aspirin, a competitive cyclooxygenase inhibitor that reduces thromboxane A_2_ synthesis and inhibits platelet aggregation. The addition of a P2Y_12_ inhibitor as a second antiplatelet agent provides further suppression of platelet function through a complementary pathway and has shown significant benefits in reducing ischemic complications in patients with CVD. Therefore, dual antiplatelet therapy (DAPT) is the cornerstone of antithrombotic therapy in several clinical settings, including ACS and ischemic stroke [5, 9, 10].

The P2Y_12_ inhibitor clopidogrel, when added to aspirin, has been extensively investigated as part of DAPT. Clopidogrel is a second-generation thienopyridine that was introduced in the United States (US) in 1998. It is administered as an inactive prodrug, with approximately 50% being absorbed through the gastrointestinal tract by the drug efflux transporter P-glycoprotein. The prodrug requires hepatic conversion via cytochrome (CYP) P450 enzymes, mainly 2C19, to produce an active metabolite. Therefore, responsiveness to clopidogrel may be diminished by concomitant administration of drugs that competitively inhibit CYP enzymes [11, 12]. In addition, interindividual variability in clopidogrel response has been observed due to multiple factors, including age, drug-drug interactions, comorbidities (e.g., diabetes or kidney failure), and genetic polymorphisms [13].

Prasugrel and ticagrelor are third-generation P2Y_12_ inhibitors that were developed to address the slow onset and heterogeneous platelet inhibitory properties of clopidogrel. In patients with ACS, randomized controlled trials (RCTs) showed a greater reduction in recurrent ischemic events with these novel agents compared with clopidogrel [14–17]. However, the newer P2Y_12_ inhibitors were associated with an increased risk of nonfatal bleeding complications, thus limiting the benefit.

Over the past few years, several interesting questions concerning the use of P2Y_12_ inhibitors have emerged. Firstly, clopidogrel was included as the comparator agent in RCTs of ticagrelor and prasugrel [14–17], and although most patients with ACS receive ticagrelor or prasugrel, clopidogrel is still widely prescribed [18]. Secondly, the use of newer generation P2Y_12_ inhibitors is associated with increased costs and a higher risk of bleeding [16, 17, 19–21], as well as nonbleeding adverse effects (e.g., dyspnea with ticagrelor use) [22]. Hence, deescalation in antiplatelet therapy (i.e., switching from the newer more potent P2Y_12_ inhibitors to clopidogrel) has become part of stage-adapted therapy [23]. Lastly, real-world studies have not confirmed the benefits of the newer P2Y_12_ inhibitors over clopidogrel with regard to efficacy and safety. For example, the CHANGE DAPT study in ACS patients treated by percutaneous coronary intervention (PCI) showed that DAPT with ticagrelor was associated with an increased risk of adverse clinical and cerebral events compared with clopidogrel [24], in contrast with the PLATO trial, which showed a decrease in these events with ticagrelor [17]. These findings may have a significant impact on DAPT use, and therefore, the exact role of clopidogrel and the newer P2Y_12_ inhibitors in different clinical settings has yet to be determined.

This review provides a comprehensive and critical discussion of the available evidence concerning antiplatelet use in patients with CVD, with a focus on the role of clopidogrel in the secondary prevention of ACS, transient ischemic attack (TIA) or minor stroke, and peripheral artery disease (PAD).

## 2. Clopidogrel in ACS: The Case for Deescalation of Therapy

The Global Registry of Acute Coronary Events (GRACE) in patients with suspected ACS showed that, despite secondary preventative therapy, 7.1% of patients died, 6.3% experienced heart failure (HF), and 4.4% experienced reinfarction during the first 2 years after ACS [6]. Current US and European guidelines recommend DAPT in patients with ACS [5, 25], and the 2018 European guidelines on myocardial revascularization recommend the use of prasugrel and ticagrelor over clopidogrel [23]. Prasugrel and ticagrelor are effective to procedural MI in the acute phase of ACS because of their ability to raise blood concentration [23].

In the last few years, thrombotic complications have decreased with the use of latest generation drug-eluting stents (DESs) and more potent P2Y_12_ inhibitors, while awareness of the impact of bleeding complications for adverse outcomes, including mortality, has increased [26]. As a result, reducing the risk of bleeding has become one of the major goals of DAPT, and guidelines recommend that the choice of treatment should consider the benefit-risk balance between the risk of ischemic and bleeding events [23]. The need for an optimal balance between ischemic benefit and bleeding risk, as well as reducing the risk of nonbleeding adverse effects as ticagrelor-related dyspnea, or/and the costs associated with long-term use of the newer P2Y_12_ inhibitors, has led to the development of DAPT “deescalation” (i.e., the switching from a more potent to a less potent P2Y_12_ inhibitor, usually clopidogrel) [23].

Deescalation has emerged as a medium- to long-term bleeding reduction strategy in patients after PCI, when thrombotic risk decreases but the bleeding risk persists, and in patients deemed unsuitable for long-term potent and more expensive antiplatelet agents (e.g., those with high bleeding risk or low socioeconomic status), and clinical trials have assessed the benefits of DAPT deescalation in patients with ACS [27, 28].

### 2.1. Platelet Function Testing

Platelet function testing (PFT) may be used to assess an individual's response to antiplatelet therapy [29, 30]. On-treatment high platelet reactivity (HPR) has been associated with an increased risk of cardiovascular events, including stent thrombosis, while low platelet reactivity (LPR) may lead to an enhanced response to P2Y_12_ inhibitors and an increased bleeding risk [29–31]. In the ARMYDA-2 study, PFT was used to assess whether a 600 mg loading dose of clopidogrel would achieve more rapid maximal platelet inhibition than a 300 mg loading dose, with a final goal of providing tailored antiplatelet therapy based on PFT results [32]. The 600 mg loading dose of clopidogrel is now the standard approach when this drug is used in patients undergoing PCI. However, the lack of the standardized PFT methodology and analytical variability may lead to misinterpretation of PFT results [33].

RCTs assessing the clinical benefit of PFT to adjust antiplatelet therapy during or early after PCI, including GRAVITAS [34], TRIGGER-PCI [35], ARCTIC [36], and ANTARCTIC [37], have failed to demonstrate the clinical benefits of PFT. One reason for the failure of these studies may be that patients with HPR were randomized to clopidogrel continuation or switching to a more potent P2Y_12_ inhibitor, despite the fact that previous studies had already demonstrated that the positive predictive value of HPR for recurrent ischemic events is low (<60%). Given the very high negative predictive value of an adequate response to clopidogrel (>90%), a more appropriate approach would be studies investigating ischemic events with clopidogrel continuation versus switching to ticagrelor in patients without HPR, with a primary analysis for noninferiority of clopidogrel continuation.

The previous version of the European guidelines on myocardial revascularization recommended limiting the use of PFT or genetic testing to specific high-risk patients (e.g., those with a history of stent thrombosis, compliance issues, suspected resistance, or a high bleeding risk) [38]. However, given the increased bleeding risk with newer antiplatelet agents and their associated adverse effect that may lead to discontinuation, RCTs have investigated alternative deescalation strategies that may include a role for PFT.

The TOPIC study investigated outcomes in patients with ACS (*n* = 646) following a switch to clopidogrel at 1 month after ACS versus continuing prasugrel or ticagrelor [27]. At 1 year after ACS, the combined endpoint of cardiovascular death, stroke, unplanned hospitalization leading to revascularization, or a Bleeding Academic Research Consortium (BARC) bleeding category ≥2 occurred in significantly more patients who continued prasugrel or ticagrelor compared with those who switched to clopidogrel [27]. Other limitations of TOPIC included its single-center and open-label design, the limited sample size, the number of patients lost to follow-up or crossing over to the other treatment arm exceeding the total number of events for many of the individual endpoints, a low-risk patient profile, no data on MI without revascularization, and the study being underpowered for stent thrombosis. Despite these limitations, TOPIC was the first RCT to evaluate a deescalation strategy not guided by PFT. Furthermore, there was no significant difference in ischemic complications at 1 year with prasugrel or ticagrelor versus clopidogrel, resulting in a net clinical benefit in favor of switching to clopidogrel-based DAPT [27].

In the TROPICAL-ACS study, PFT-guided DAPT deescalation (early switch from prasugrel to clopidogrel) was noninferior to prasugrel at 1 year with regard to the risk of cardiovascular death, myocardial infarction (MI), or stroke (referred to hereafter as major adverse cardiovascular events (MACEs)) after PCI for ACS [28]. This study was important as it represented a comparison of no PFT (newer P2Y_12_ inhibitor) versus a PFT-guided strategy. However, some limitations of TROPICAL-ACS were the fact that 40% of patients in the deescalation group required escalation back to prasugrel (thereby nullifying any bleeding advantage) and that it is difficult to replicate this study in clinical practice, as there were two therapeutic changes in 2 weeks. Furthermore, no clopidogrel loading dose was used, no mention of transition events is provided, a higher than expected proportion of patients on prasugrel had HPR (15%), and data according to the type of antiplatelet therapy in the PFT-guided arm were not available [28]. Evidence was provided for considering HPR a modifiable risk factor, with HPR on prasugrel being associated with an increased risk for ischemic events and LPR being an independent predictor of bleeding both with prasugrel and with clopidogrel.

Based on evidence from these studies, recent clinical guidelines recommend DAPT deescalation as a strategy that may be considered an alternative treatment option for ACS patients. The 2018 European guidelines on myocardial revascularization now recommend considering a PFT-guided DAPT deescalation strategy as an alternative DAPT strategy, particularly in patients with ACS in whom 12 months of potent antiplatelet therapy may not be appropriate [23]. Furthermore, a recent consensus statement supports PFT- or genotype-guided deescalation, although these experts stated that, in patients undergoing PCI, PFT-guided deescalation may only be considered in specific clinical scenarios [39].

### 2.2. Genotype Testing

A recent RCT has investigated the benefits of genotype-guided selection of antiplatelet therapy in patients undergoing primary PCI with stent implantation (*n* = 2488) [40]. In this study, patients were assigned to receive P2Y_12_ inhibitor therapy based on early CYP2C19 genetic testing (genotype-guided group) or either ticagrelor or prasugrel (standard-treatment group). Over 12 months, genotype-guided therapy was noninferior to standard therapy with regard to the combined net adverse clinical outcome of death from any cause, MI, definite stent thrombosis, stroke, or PLATO major bleeding (5.1% versus 5.9%; 95% CI, −2.0 to 0.7; *P* < 0.001 for noninferiority). However, the risk of the primary bleeding outcome was significantly reduced with genotype-guided therapy versus standard treatment (9.8% versus 12.5%; hazard ratio (HR), 0.78; 95% CI, 0.61 to 0.98; *P*=0.04). Of note, CYP2C19 genotyping in this study was performed using central laboratory assays or an on-site point-of-care device [40], which represent quick and easy methods for genotype-guided selection of oral P2Y_12_ inhibitors [41]. Furthermore, a personalized pharmacogenomic approach to selecting antiplatelet therapy for patients with ACS on the basis of a patient's genetic (such as CYP2C19) and clinical characteristics may reduce ischemic and bleeding events [42]. In addition, ethnic and racial variability in drug metabolism is also known to contribute to the polymorphic expression of metabolizing enzymes [41]. The benefits of testing CYP2C19 polymorphisms before prescribing clopidogrel in patients treated with drug-eluting stent implantation after PCI have been suggested by some studies, mainly in Asian populations [43]. However, genetic polymorphisms can explain only 12% of clopidogrel response variability [44], as suggested by the suboptimal concordance between the genotype and the phenotype ARCTIC-Gene substudy [45].

### 2.3. DAPT Deescalation in High-Risk Patients

In a real-world study of Italian patients with ACS and diabetes (*n* = 559), DAPT was prescribed at hospital discharge in 88% of the patients (39%, 38%, and 23% received clopidogrel, ticagrelor, and prasugrel, respectively) [46]. The authors concluded that this confirmed the “paradox” of using a less effective drug to treat sicker patients in this high-risk population [46]. However, the features of increased ischemic risk may also predict a higher bleeding risk, which may also explain the prevalent use of clopidogrel. The presence of diabetes has been shown to increase the risk of ischemic events but also significantly increases the risk of bleeding complications. Thus, data from this real-world study suggest that physicians use “very early deescalation” by prescribing at hospital discharge the medication they consider the best option to manage the thrombosis-bleeding risk trade-off in these high-risk patients.

Bleeding risk is of particular concern in elderly patients, who represent a large proportion of patients with ACS; however, this patient population was underrepresented in the PLATO and TRITON trials [16, 17]. In the recently presented POPular AGE study of patients aged ≥70 years with NSTE-ACS, after 12 months, treatment adherence was 76% with clopidogrel versus 51% with ticagrelor [47]. The most common reasons for discontinuation of ticagrelor were bleeding, initiation of oral anticoagulation, and dyspnea. The relative risk of major or minor bleeding was significantly reduced by 26% with clopidogrel, with PLATO major bleeding reported in 4.4% of patients with clopidogrel versus 8% with ticagrelor or prasugrel. The net clinical benefit (defined as the composite of all-cause mortality, MI, stroke, or PLATO major or minor bleeding) showed an absolute risk difference of 3.4% in favor of clopidogrel, which did not reach the prespecified cutoff for noninferiority [47]. Similarly, the Elderly ACS 2 trial in patients aged >74 years with ACS undergoing PCI was prematurely terminated after a planned interim analysis found no significant difference between reduced-dose prasugrel and standard-dose clopidogrel with regard to the primary endpoint (composite of death, MI, disabling stroke, or rehospitalization for cardiovascular causes or bleeding) [48]. In this study, the rate of BARC bleeding >2 was similar between prasugrel and clopidogrel (4.1% versus 2.7%; odds ratio (OR), 1.52; 95% CI, 0.85 to 3.16; *P*=0.18) [48]. Although data from the Elderly ACS 2 trial should be interpreted with caution due to its premature termination, they suggest that there is no difference in efficacy and safety between prasugrel and clopidogrel in elderly patients with ACS.

## 3. Clopidogrel in ACS: Real-World Studies

Although RCTs are considered the gold standard of clinical research, RCT participants often differ from patients treated in routine clinical practice, which may limit the generalizability of RCT results. Therefore, an increasing number of postauthorization (phase IV), real-world studies of antiplatelet therapy in patients with ACS have been conducted.

The PIRAEUS group integrated data from 10 European ACS registries, to gain a comprehensive overview on the efficacy and safety of the P2Y_12_ inhibitors in patients with STEMI and non-ST-elevation ACS (NSTE-ACS) during real-life clinical practice [49–51]. Patients' characteristics and main outcomes of patients with NSTE-ACS and STEMI treated with DAPT showed similar rates of mortality, ischemic events, and bleeding events than those reported in RCTs of the various P2Y_12_ inhibitors. Yet, important differences in use and patient selection between clopidogrel, prasugrel, and ticagrelor were found. All registries documented a large number of patients on clopidogrel, with fewer patients on prasugrel, and ticagrelor use was recorded only in a limited number of registries. Moreover, clopidogrel was administered in older and sicker patients [51]. Although the comparability of results is limited by differences between registries in the study setting, endpoint definitions, and patient selection, PIRAEUS highlights the importance of standardized data collection to enable more robust common analyses of multiple registries [50, 51].

The PROMETHEUS registry study enrolled patients with ACS undergoing PCI at eight centers in the US to determine the frequency of prasugrel use and its association with clinical outcomes in this patient population [52]. Prasugrel use was associated with a significantly lower rate of MACEs (HR, 0.58; 95% CI, 0.50 to 0.67; *P* < 0.001) and bleeding (HR, 0.65; 95% CI, 0.51 to 0.83; *P* < 0.001) at 90 days compared with clopidogrel. However, these associations were attenuated and no longer significant after propensity stratification, as patients receiving prasugrel were generally younger and presented with fewer comorbidities than those receiving clopidogrel [52].

The GRAPE registry study investigated the long-term efficacy and safety of clopidogrel, prasugrel, and ticagrelor in real-world acute ACS patients who underwent PCI [53]. After 1 year of follow-up, the rate of MACEs was lower with prasugrel versus clopidogrel (4.4% versus 10.1%; HR, 0.53; 95% CI, 0.30 to 0.91) but was similar with ticagrelor and clopidogrel (6.8% versus 10.1%; HR, 0.78; 95% CI, 0.54 to 1.12). Compared with clopidogrel, the risk of any type of BARC-classified bleeding was higher with prasugrel (HR, 1.61; 95% CI, 1.33 to 1.95) and ticagrelor (HR, 1.81; 95% CI, 1.55 to 2.10). An adjusted comparison showed no difference in any outcomes between prasugrel- and ticagrelor-treated patients. This study concluded that, in PCI-treated patients with ACS, prasugrel showed better anti-ischemic benefits over clopidogrel, although the use of prasugrel and ticagrelor was associated with an increased risk of bleeding events [53].

Of note, differences in baseline patient characteristics between the three P2Y_12_ inhibitor groups should be considered when interpreting the results of the GRAPE registry study [53]. Risk factors for ischemic or bleeding complications were more common among patients in the clopidogrel group than those receiving prasugrel or ticagrelor (i.e., they were older, higher proportions were female, and they had a history of hypertension, prior stroke, or impaired renal function) [53]. Similar patient selection biases were previously reported in real-world studies comparing clopidogrel with other P2Y_12_ inhibitors [54–56]. Furthermore, in the SWEDEHEART registry study of ACS patients treated with or without PCI, mortality rates were lower with ticagrelor versus clopidogrel, but significantly more patients on ticagrelor were treated with PCI and ticagrelor was preferentially used in patients with a low risk of bleeding and death (as indicated by lower CRUSADE and GRACE scores, respectively) [57, 58].

Current guidelines recommend the use of ticagrelor over clopidogrel in patients with ACS, mainly based on the results of the randomized PLATO trial [17]. In PLATO, ticagrelor significantly reduced the risk of MACEs by 16% at 12 months compared with clopidogrel (HR, 0.84; 95% CI, 0.77 to 0.92; *P* < 0.001) but was associated with an increased rate of noncoronary artery bypass graft-related major bleeding (4.5% versus 3.8%; *P*=0.03) [17].

Notably, more than 60% of patients in PLATO who underwent PCI received bare metal stents (BMSs), and most DESs were first-generation devices [17]. Since newer generation DESs have become available, with thinner stent struts covered by more biocompatible or biodegradable polymer coatings, clinical outcomes have improved compared with BMSs and first-generation DESs [59–61]. Thus, in clinical practice, most patients with ACS are treated with newer generation DESs that have shown favorable results with clopidogrel-based DAPT in RCTs [62, 63].

The real-world CHANGE DAPT study evaluated the safety and efficacy of a ticagrelor- versus clopidogrel-based DAPT regimen in ACS patients treated with newer generation DESs [24]. In propensity score-adjusted multivariate analysis, ticagrelor was associated with an increased risk of the composite endpoint of net adverse clinical and cerebral events (defined as all-cause death, any MI, stroke, or major bleeding; HR, 1.75; 95% CI, 1.20 to 2.55; *P*=0.003) and major bleeding (HR, 2.75; 95% CI, 1.34 to 5.61; *P*=0.01) compared with clopidogrel [24]. These results are consistent with those of the GRAPE registry [53]. Moreover, in CHANGE DAPT, the increased bleeding risk with ticagrelor was observed despite more transradial procedures, more pump inhibitor use, and less glycoprotein IIb/IIIa inhibitor use, factors which may reduce periprocedural bleeding [24]. These data are also consistent with the TOPIC trial, in which switching from prasugrel or ticagrelor to clopidogrel 1 month after PCI was not associated with significant changes in ischemic outcomes but resulted in fewer bleeding events [27]. Therefore, real-world studies do not confirm the superior efficacy of newer P2Y_12_ inhibitors over clopidogrel in ACS patients treated with PCI, and new research on this topic is warranted.

As well as being effective and safe in patients with ACS, studies have indicated that clopidogrel is cost effective in this patient population, with an estimated cost-effectiveness ratio of approximately $3,000 per life year gained [64, 65]. Therefore, according to World Health Organization criteria, clopidogrel is cost effective in countries with a gross domestic product of more than $1,000 per capita [66].

## 4. Clopidogrel in Transient Ischemic Attack and Acute Stroke

A characteristic of TIA and minor ischemic strokes is a rapid recovery from the symptoms of cerebral ischemia [67, 68]. This rapid clinical recovery may indicate the presence of at-risk ischemic tissue, a pathophysiologic trait that may be responsible for greater instability [68, 69]. Therefore, although TIA and minor stroke do not cause disabling symptoms, they often precede a more severe, disabling stroke, or other vascular events [70, 71]. A systematic review and meta-analysis found that the risk of stroke was 17% in the 90 days following a TIA [71], and in a population-based database study, the combined risk of stroke, MI, or death was 22% over a 1-year follow-up after TIA [72]. A more recent TIA registry study showed that the risk of recurrent TIA or stroke remained similar over 1–5 years after the index event [73].

Early initiation of antiplatelet treatment is recommended for patients with noncardioembolic stroke or TIA to prevent recurrent stroke or cardiovascular events. In the population-based EXPRESS study, early treatment after TIA was associated with an 80% reduction in the 90-day risk of recurrent stroke [74]. In another study, the early risk of recurrent stroke was significantly lower in patients who received rapid TIA assessment and treatment compared with standard care (9.7% versus 4.7%; *P*=0.05) [75].

Aspirin is the most common antiplatelet agent used to treat patients with a history of TIA or stroke as it reduces the risk of stroke recurrence. RCTs have demonstrated that DAPT may also be effective in these patients [76–79]. However, until recently, Italian guidelines stated that DAPT has to be considered only for selected high-risk TIA and minor stroke patients and for a short period (2-3 weeks) after stroke onset [80].

### 4.1. DAPT for Secondary Prevention of TIA or Stroke

Several RCTs have investigated the efficacy and safety of DAPT for secondary prevention in patients with a history of TIA or stroke.

In the MATCH trial of 7,599 patients with a recent history of TIA or stroke, aspirin plus clopidogrel did not significantly reduce the risk of the composite primary endpoint of ischemic stroke, MI, worsening of peripheral arterial disease, vascular death, or rehospitalization for acute ischemia compared with placebo plus clopidogrel over 18 months (relative risk reduction, 6.4%; 95% CI, −4.6 to 20.4; *P*=0.244) [78]. However, the incidence of life-threatening bleeding was higher with aspirin plus clopidogrel versus clopidogrel alone (2.6% versus 1.3%; difference, 1.3%; 95% CI, 0.6 to 1.9; *P* < 0.0001). Therefore, this study showed that adding aspirin to clopidogrel in high-risk patients did not significantly reduce major vascular events and was associated with an increased risk of major bleeding [78]. Moreover, bleeding complications remained constant over the study duration, which may suggest that there is a time margin after which the risk of bleeding might outweigh any ischemic benefit.

In the CHANCE trial of 5,170 Chinese patients with nondisabling ischemic stroke or TIA, clopidogrel plus aspirin for 21 days followed by clopidogrel alone for 69 days (DAPT) reduced the risk of recurrent ischemic and hemorrhagic stroke compared with aspirin alone by 32% (8.2% versus 11.7%; HR, 0.68; 95% CI, 0.57 to 0.81; *P* < 0.001) [81]. DAPT was associated with similar rates of moderate or severe bleeding (0.3% in each group; *P*=0.73) or hemorrhagic stroke (0.3% in each group; *P*=0.98) versus aspirin alone [81]. Interestingly, the clopidogrel plus aspirin group continued to have a significantly lower risk of stroke after 1 year of follow-up (HR, 0.78; 95% CI, 0.65 to 0.93; *P*=0.006) [82]. These findings indicate that DAPT with aspirin plus clopidogrel, initiated within 24 hours of the index event, is superior to aspirin alone for preventing the risk of stroke, without increasing the risks of hemorrhage in patients with TIA or minor stroke [81].

The generalizability of the CHANCE results may be questioned as the study was conducted entirely in China, in a population with a higher incidence of large-artery intracranial atherosclerosis than in other countries. In addition, CHANCE screened 41,561 patients with stroke or TIA to find 5,170 (12.4%) appropriate subjects to enroll, and patients with major ischemic stroke, who are at risk for hemorrhagic transformation, were excluded [81]. Finally, the results of this trial cannot be generalized beyond 90 days after the index event because thereafter the cumulative risk of bleeding with clopidogrel plus aspirin compared with aspirin alone offsets the benefits, as shown in earlier studies [77, 78, 83].

The POINT trial compared the safety and efficacy of clopidogrel plus aspirin versus aspirin alone in a non-Chinese population of 4,881 patients with nondisabling ischemic stroke or TIA [84]. Within 12 hours of symptom onset, patients were randomized to receive either clopidogrel (600 mg loading dose followed by 75 mg daily) plus aspirin (50–325 mg daily) or aspirin alone for 90 days. Clopidogrel plus aspirin was associated with a significantly lower risk of major ischemic events (ischemic stroke, MI, or ischemic vascular death) compared with aspirin alone (5.0% versus 6.5%; HR, 0.75; 95% CI, 0.59 to 0.95; *P*=0.02) and a higher risk of major hemorrhage at 90 days (0.9% versus 0.4%; HR, 2.32; 95% CI, 1.10 to 4.87; *P*=0.02) [84]. This higher risk of major hemorrhage was likely related to the longer duration of clopidogrel plus aspirin therapy and the high initial loading dose of clopidogrel (600 mg) used in the POINT trial. Notably, the findings of POINT confirm and expand the results of the CHANCE trial, supporting the hypothesis that the effective use of DAPT for early secondary stroke prevention is related to ethnicity [84].

In a prespecified secondary analysis of POINT, the rate of primary efficacy events with clopidogrel plus aspirin was 3.6% during 0–21 days and 1.4% during 22–90 days versus 5.6% during 0–21 days and 0.9% during 22–90 days with aspirin alone [85]. Conversely, the rate of major hemorrhage remained constant in both groups during the 90 days (0.4% during 0–21 days and 0.5% during 22–90 days with clopidogrel plus aspirin versus 0.2% during 0–21 days and 0.2% during 22–90 days with aspirin alone) [85]. These results, coupled with the findings of the CHANCE trial, indicate that the optimal duration of DAPT (clopidogrel plus aspirin) is 21–28 days. Moreover, the results of CHANCE suggest that, after the first phase of DAPT (22–90 days), clopidogrel alone is more effective than aspirin alone when compared from days 22 to 90, without an increased risk of bleeding [81]. A recent metaregression of 11 RCTs and 24,175 patients showed that the greatest benefit of DAPT in terms of prevention of recurrent stroke was observed in patients with a more elevated risk profile at baseline, increased stroke severity, or concurrent carotid artery disease and in patients who received early initiation of DAPT for ≤3 months [86].

When considering the effect of newer P2Y_12_ inhibitors, the SOCRATES trial found that ticagrelor was not superior to aspirin in reducing the risk of stroke, MI, or death at 90 days in patients with acute ischemic stroke or TIA [87]. Although there was no significant difference in the rate of serious adverse events between groups, permanent discontinuation was more common with ticagrelor, mainly due to dyspnea (a known adverse effect of ticagrelor [17, 88]) [87]. Interestingly, in a meta-analysis of 12 RCTs of aspirin versus control in the secondary prevention after TIA or ischemic stroke (*n* = 15,778), aspirin reduced the 6-week risk of recurrent ischemic stroke by 58% (HR, 0.42; 95% CI, 0.32 to 0.55; *P* < 0.0001) and disabling or fatal ischemic stroke by 71% (HR, 0.29; 95% CI, 0.20 to 0.42; *P* < 0.0001), but these benefits diminished with longer term use [89]. These data support the need for more intensive antiplatelet therapy (DAPT) in the early postevent period, when the ischemic risk is higher, and less intensive treatment thereafter to minimize the risk of bleeding complications.

The 2018 American Heart Association/American Stroke Association guidelines recommend the use of DAPT (aspirin and clopidogrel) for 21 days in patients with minor stroke (class of recommendation IIa, level of evidence B-R) [10], and the 2018 update of the Canadian Stroke guidelines suggests DAPT with clopidogrel plus aspirin for 21–30 days followed by monotherapy with aspirin or clopidogrel alone in very high-risk patients with TIA (ABCD2 score > 4) or minor stroke of noncardioembolic origin (evidence level A) [90]. In 2018, the Italian Stroke Organization (ISO)-Stroke Prevention and Educational Awareness Diffusion (SPREAD) working group recommended DAPT with aspirin plus clopidogrel for 30 days in patients with minor stroke or TIA [91].

## 5. Clopidogrel in Peripheral Artery Disease

Peripheral artery disease (PAD) is characterized by the narrowing or blockage of the arteries of the lower extremities due to atherosclerosis. The term “peripheral arterial diseases” encompasses all atherosclerotic diseases in arteries other than the coronary arteries and aorta [92]. PAD is a global health issue, with high levels of associated morbidity and mortality and an estimated overall prevalence of 3–10%, and 15–20% in those aged >70 years [93]. This burden is expected to increase significantly during the next 20 years, due to population aging and changes in atherosclerosis risk factors. Over a 10-year period (2000–2010), PAD was notably more prevalent in low- or middle-income countries than in high-income countries [94].

The risk factors for PAD include older age, diabetes, hyperlipidemia, hypertension, smoking, and atherosclerosis at other sites [95]. PAD is usually asymptomatic in the initial clinical stage. The most common first symptom is intermittent claudication (IC), defined as lower limb pain induced by physical activity that is rapidly relieved at rest [93]. Disease progression may result in critical limb ischemia (CLI), defined as pain at rest or ischemic ulceration and gangrene [93], which is associated with severe impairment of lower limb function and a high risk of amputation, especially in patients who cannot undergo a surgical or endovascular revascularization [96]. In addition, patients with PAD typically exhibit multivessel disease and may also present with coronary artery disease (CAD) or cerebral artery disease, which further reduces their quality of life [97]. Patients with symptomatic or asymptomatic PAD have an increased risk of all-cause mortality, cardiovascular mortality, MI, and stroke, even after adjustment for conventional risk factors [92, 95].

The aim of PAD management is to alleviate symptoms and prevent disease progression and complications [92]. Medical treatment includes lifestyle modifications, such as dietary changes and increased physical activity, and risk-factor modification, such as smoking cessation and the initiation of antihypertensive and lipid-lowering drugs [92]. As cardiovascular risk factors can lead to the development of atherosclerosis and atherothrombosis due to platelet activation [98, 99], antiplatelet therapy in addition to risk-factor modification is the hallmark treatment to reduce cardiovascular events in patients with PAD [92].

### 5.1. Antiplatelet Therapy for PAD

The 2017 European Society of Cardiology (ESC)/European Society for Vascular Surgery (ESVS) guidelines stated that long-term single antiplatelet therapy is recommended in symptomatic PAD patients (class of recommendation I, level of evidence A) and in all patients who have undergone revascularization (class of recommendation I, level of evidence C) [92]. In both cases, clopidogrel may be preferred over aspirin (class of recommendation IIb, level of evidence B) [92]. However, antiplatelet therapy is not routinely indicated in patients with isolated asymptomatic PAD because of a lack of proven benefit.

These recommendations are based, at least in part, on the results of the CAPRIE trial [100]. In this study of 19,185 patients with a history of MI, ischemic stroke, or symptomatic PAD, the relative risk of the primary outcome (MACEs) was significantly reduced with clopidogrel versus aspirin (5.3% versus 5.8%; relative risk reduction, 8.7%; 95% CI, 0.30 to 16.5; *P*=0.043) [100]. Although these results suggested that long-term clopidogrel therapy may be superior to aspirin in reducing the risk of vascular events, these benefits were marginal. However, the benefit of clopidogrel over aspirin was mainly driven by the large effect shown in patients with PAD, raising the possibility that clopidogrel and aspirin had equivalent efficacy in patients presenting with MI. In the subgroup of patients with symptomatic PAD at baseline (*n* = 6,452), clopidogrel was associated with a 22% reduction versus aspirin in the relative risk of MACEs (HR, 0.78; 95% CI, 0.65 to 0.93), as well as a significant reduction in the risk of cardiovascular death (HR, 0.76; 95% CI, 0.64 to 0.91). Both treatment groups had comparable rates of major bleeding [100].

In the CHARISMA trial, DAPT with clopidogrel plus aspirin was not more effective than aspirin monotherapy in preventing the primary outcome of MACEs in patients with stable atherosclerotic disease or multiple cardiovascular risk factors (*n* = 15,603) [77]. A post hoc analysis of CHARISMA participants with PAD (*n* = 3,096) showed that the primary outcome occurred at a similar rate with clopidogrel plus aspirin versus aspirin monotherapy (7.6% versus 8.9%; HR, 0.85; 95% CI, 0.66 to 1.08; *P*=0.18) [101]. However, DAPT reduced the risk of other secondary endpoints, such as MI (HR, 0.63; 95% CI, 0.42 to 0.96; *P*=0.029) and the rate of hospitalization for ischemic events (HR, 0.81; 95% CI, 0.68 to 0.95; *P*=0.011). There was an increased rate of minor bleeding with clopidogrel plus aspirin versus aspirin alone (OR, 1.99; 95% CI, 1.69 to 2.34; *P* < 0.001), although the rates of severe, fatal, or moderate bleeding did not differ between the groups [101].

In a post hoc analysis of the PLATO trial [17], patients with coronary disease and concurrent PAD showed some ischemic benefit with ticagrelor versus clopidogrel [102], and in the PEGASUS-TIMI 54 trial of patients with prior MI (*n* = 21,162), those with concurrent PAD (*n* = 1,143) showed a significantly greater reduction in the absolute risk of MACEs with ticagrelor compared with patients without PAD [103].

Most studies investigating the effect of antiplatelet treatment in high-risk atherothrombotic diseases have focused on patients with ACS and stable CAD. The EUCLID trial was designed to evaluate antiplatelet therapies with ticagrelor versus clopidogrel in patients with symptomatic PAD (*n* = 13,885) [104]. In this trial, the incidence of the primary efficacy endpoint (MACEs) was similar with ticagrelor and clopidogrel (10.8% versus 10.6%; HR, 1.02; 95% CI, 0.92 to 1.13; *P*=0.65), and the primary safety endpoint (major bleeding) occurred in 1.6% of the patients in both groups (HR, 1.10; 95% CI, 0.84 to 1.43; *P*=0.49). The incidences of acute limb ischemia and revascularization were similar between groups, whereas the relative risk of ischemic stroke was significantly reduced with ticagrelor versus clopidogrel (1.9% versus 2.4%; HR, 0.78; 95% CI, 0.62 to 0.98; *P*=0.03). There were fewer fatal bleeding events with ticagrelor but more discontinuations of ticagrelor than clopidogrel, including discontinuations due to bleeding [104]. Hence, despite showing some benefit in patients with PAD in earlier studies, monotherapy with ticagrelor, a more potent P2Y_12_ inhibitor than clopidogrel, failed to demonstrate any benefit over clopidogrel monotherapy in reducing the rate of adverse cardiovascular events in the EUCLID study and showed a similar rate of major bleeding.

Interestingly, the COMPASS trial of rivaroxaban use (with or without aspirin) in patients with stable CVD [105] may help to enlighten our understanding of the role of antiplatelet and antithrombotic strategies in patients with PAD. In COMPASS, which included patients with established CAD, PAD, or both, the primary efficacy endpoint (MACEs) occurred in 4.1% of patients in the rivaroxaban plus aspirin group, 4.9% in the rivaroxaban monotherapy group, and 5.4% in the aspirin monotherapy group, representing a 24% reduction in the relative risk of MACEs with low-dose rivaroxaban plus aspirin versus aspirin alone (HR, 0.76; 95% CI, 0.66 to 0.86; *P* < 0.001) [105]. Rivaroxaban plus aspirin was also associated with a reduction in all-cause mortality compared with aspirin alone (3.4% versus 4.1%; HR, 0.82; 95% CI, 0.71 to 0.96; *P*=0.01). In contrast, rivaroxaban alone was associated with a significant reduction in the risk of MACEs versus aspirin alone (HR, 0.90; 95% CI, 0.79 to 1.03; *P*=0.12). More major bleeding events were reported with either rivaroxaban plus aspirin (3.1%) or rivaroxaban monotherapy (2.8%) than with aspirin monotherapy (1.9%; *P* < 0.001 for both comparisons) [105]. In a prespecified analysis of patients with PAD from the COMPASS trial (*n* = 7,470), there were a 28% reduction in the risk of MACEs, a 46% reduction in the risk of major adverse limb events (MALEs), and a 70% reduction in the risk of major amputations with rivaroxaban plus aspirin versus aspirin alone [106]. However, increased rates of major and minor bleeding were observed with rivaroxaban plus aspirin compared with aspirin monotherapy.

The intriguing question arising from a critical analysis of the COMPASS trial results is why was aspirin chosen as the comparator in this trial? Considering that almost one-third of patients in the study had PAD and given the somewhat contradictory evidence in favor of aspirin in this clinical setting [107] as compared with that of clopidogrel in studies such as CAPRIE [100], different results may hypothetically be expected from a comparison between rivaroxaban and clopidogrel. Nevertheless, given the limits of indirect comparisons and the differences in the design of the aforementioned studies, these data suggest that, in patients with PAD, the safety of clopidogrel alone may be better than that of rivaroxaban plus aspirin, with comparable efficacy with regard to MACEs.

## 6. Conclusions

Deescalation from ticagrelor or prasugrel to clopidogrel is recommended in ACS patients to obtain an optimal balance between ischemic benefit and bleeding risk and to reduce the risk of adverse effects (such as dyspnea) and/or the increased costs associated with long-term use of newer P2Y_12_ inhibitors. Genotype-guided DAPT deescalation may be favored. Moreover, clopidogrel may be considered the first choice of antiplatelet therapy in elderly patients with ACS. The results of real-world studies have questioned the superior efficacy of newer P2Y_12_ inhibitors over clopidogrel for ACS patients treated by PCI.

In patients with stroke or very high-risk TIA, intensive DAPT with aspirin plus clopidogrel should be administered for 21–28 days after the acute event, followed by less intensive treatment for up to 90 days, to minimize the risk of bleeding complications; clopidogrel is potentially more effective than aspirin as antiplatelet monotherapy. In patients with symptomatic PAD, or those who have undergone peripheral revascularization, clopidogrel is the preferred agent for antiplatelet monotherapy based on the results of the CAPRIE and EUCLID trials.

In conclusion, given the strong evidence supporting the efficacy, safety, and cost-effectiveness of clopidogrel for antiplatelet therapy in several different clinical settings, its familiarity in the medical community, its wide availability, and low cost, clopidogrel remains an important medication in clinical practice and a mainstay of antiplatelet therapy.
